# Funmap2: an R package for QTL mapping using longitudinal phenotypes

**DOI:** 10.7717/peerj.7008

**Published:** 2019-05-31

**Authors:** Nating Wang, Tinyi Chu, Jiangtao Luo, Rongling Wu, Zhong Wang

**Affiliations:** 1 College of Biological Sciences and Technology, Beijing Forestry University, Beijing, China; 2 Graduate field of Computational Biology, Cornell University, Ithaca, NY, United States of America; 3 Department of Biostatistics, College of Public Health, University of Nebraska Medical Center, Omaha, NE, United States of America; 4 Baker Institute for Animal Health, College of Veterinary Medicine, Cornell College, Ithaca, NY, United States of America

**Keywords:** QTL mapping, Quantitative trait loci, Functional mapping, Longitudinal traits, Log-likelihood ratio test

## Abstract

Quantitative trait locus (QTL) mapping has been used as a powerful tool for inferring the complexity of the genetic architecture that underlies phenotypic traits. This approach has shown its unique power to map the developmental genetic architecture of complex traits by implementing longitudinal data analysis. Here, we introduce the R package *Funmap2* based on the functional mapping framework, which integrates prior biological knowledge into the statistical model. Specifically, the functional mapping framework is engineered to include longitudinal curves that describe the genetic effects and the covariance matrix of the trait of interest. *Funmap2* chooses the type of longitudinal curve and covariance matrix automatically using information criteria. *Funmap2* is available for download at https://github.com/wzhy2000/Funmap2.

## Introduction

Advances in sequencing technologies have dramatically increased the number of molecular markers available for studying an organism’s genome. QTL mapping exploits these markers to identify the genomic regions associated with the quantitative traits within an inbred population. In the past 20 years, a variety of statistical models have been developed to detect QTLs, which has greatly facilitated the identification of the genomic regions that control biological traits. In addition to the study of additive and dominant effects, QTL mapping has been applied successfully to the study of epistasis effects, allometric growth, and pleiotropic effects.

Lander & Botstein (1989) established tractable statistical methodologies to map QTLs on one chromosomal interval bracketed by two flanking markers, which is known as the interval mapping method. Later, composite interval mapping improved interval mapping by including markers from other intervals as covariates to control the overall genetic background (Zeng, 1994). Kao, Zeng & Teasdale (1999) proposed the simultaneous use of multiple marker intervals to map multiple QTLs of epistatic interactions throughout a linkage map. Since then, mapping QTLs in complicated genetic and genomic problems has increased dramatically.

Even though there have been advances in mapping resolution and extension to more complicated mapping problems, conventional mapping approaches are restricted to phenotypic data measured at a single point in time. In many important biological problems, however, genotypes that control longitudinal traits, such as those measured during developmental processes and environmental changes, cannot be accommodated effectively under the framework of single trait QTL mapping. Several approaches (Ma, Casella & Wu, 2002; Wu et al., 2002; Yang, Tian & Xu, 2006; Kwak et al., 2014) have been developed for QTL mapping of such function valued traits. Functional mapping (Ma, Casella & Wu, 2002; Wu & Lin, 2006; Sun et al., 2015) is a statistical framework derived to map genes that control the dynamic biological process of complex traits. In this framework, a mixture model is fitted using an EM algorithm by maximizing likelihood, followed by hypothesis testing of the significance of association. In addition, model parameters that describe growth trajectories can also be estimated. Functional mapping has shown remarkable performance in associating QTLs with dynamic traits in plants (Zhao et al., 2004b; Li et al., 2010b; Yang et al., 2011, Sillanpää et al., 2012), animals (Zhao et al., 2004a; Xiong et al., 2011), and humans (Li, Das & Wu, 2009). Its application can be extended to genetic dissection of developmental processes that include growth trajectory and allometric scaling (Ma et al., 2003; Li et al., 2014), phenotypic plasticity based on gene-environment interaction (Wang et al., 2013a), drug response (Wang et al., 2013b), and morphological shape (Fu et al., 2013). Recently, the integration of functional mapping and differential equations (Fu et al., 2011; Wang et al., 2013b) has also been applied to widely emergent applications of dynamic systems.

The *Funmap2* package is developed to identify QTLs for a longitudinal trait based on functional mapping. It is implemented as a package for the freely accessible statistical software R (R Core Team, 2019). *Funmap2* implements a complete pipeline, which includes data loading, QTL scanning, computing of significance values, and reporting of significant QTL. The essence of functional mapping relies on the longitudinal curve of genetic effects and the covariance matrix that characterize the longitudinal relationship of the trait. Although a logistic curve may describe the genetic effects in most biological processes, the reaction norm in continuously varying environmental problems may not follow a sigmoid shape. To address this, *Funmap2* provides sigmoid, Legendre, and Pharmacology as built-in curves, and it also allows users to customize the curve equation. Furthermore, to increase statistical power, the covariance matrix can be chosen from several covariance structures used in IBM SPSS software, such as autoregressive, ante-dependence, or autoregressive moving average (Li et al., 2010a). Because of the difficulty of knowing which combination of curve shapes and covariance structures is the best for a longitudinal trait beforehand, we enabled *Funmap2* to choose the best curve shape and covariance structure automatically from the built-in resources based on information criteria.

In addition to statistical analysis, *Funmap2* generates a PDF report that visualizes all results, which include phenotype traits, QTL profiles, significant QTL curves, and permutation results. Additionally, the package provides a simulation module for testing the performance and demonstrating the use of *Funmap2* on data generated by different models.

*Funmap2* is a QTL mapping tool for longitudinal traits with an open source software license. It is available publicly under an open-source software license: https://github.com/wzhy2000/Funmap2. In the following sections, we give a brief review of functional mapping in terms of its statistical model. Then, we focus on the detailed workflow of *Funmap2*. Lastly, we provide one example with codes and figures.

## Materials & Methods

### Statistical methods

Functional mapping is a statistical framework aimed at identifying QTLs that are associated significantly with a longitudinal phenotype of interest in an experimental population, such as recombinant inbred lines (RIL) or a doubled-haploid (DH) population. Functional mapping computes maximum likelihood estimation (MLE) of mixture models that integrate the likelihood over QTL genotypes. The model also accounts for the (1) longitudinal trend of the trait using a continuous curve (i.e., growth trajectory for time-dependent traits and reaction norm for environment-dependent traits), and (2) internal correlation of traits across longitudinal measurements using a covariance matrix.

The model assumes that the longitudinal trend of the trait follows a particular mathematical curve. In a statistical setting, the phenotypes of the trait at all time points follow a multivariate normal distribution, which can be described by the following Eq. (1):

(1) \begin{eqnarray*}{\mathrm{f}}_{\mathrm{j}} \left( {\mathrm{y}}_{\mathrm{i}} \right) = \frac{1}{(2\pi )^{ \frac{\mathrm{m}}{2} }{ \left\vert \sum \right\vert }^{ \frac{1}{2} }} \exp \nolimits \left[ -{ \left( {\mathrm{y}}_{\mathrm{i}}-\alpha {\mathrm{X}}_{\mathrm{i}}-{\mathrm{g}}_{\mathrm{j}} \right) }^{\mathrm{T}}{\sum \nolimits }^{-1}({\mathrm{y}}_{\mathrm{ i}}-\alpha {\mathrm{X}}_{\mathrm{i}}-{\mathrm{g}}_{\mathrm{j}})/2 \right] .\end{eqnarray*}

Assuming that y_i_ is a vector of measured values for individual *i* at *m* time points, which describes the phenotypic values, g_j_ denotes the overall mean vector for genotype *j* that is described as a mathematical curve, X_i_ denotes the covariate for individual *i*, *α* is a vector of coefficient value for each covariate, and∑ is the covariance matrix. Therefore, ${\mathrm{f}}_{\mathrm{j}} \left( {\mathrm{y}}_{\mathrm{i}} \right) $ is the probability density function that relates the measured traits of individual *i* to the combination of the genetic effects contributed by genotype *j* and covariate effects of individual *i.* In Functional mapping, we assume the genetic effects of each genotype vary from time to time and follow a trajectory which can be defined by a mathematical curve (g_j_), such as the logistic, Legendre or other type curves (Ma, Casella & Wu, 2002). For the logistic curve, (g_j_) can be described by

(2) \begin{eqnarray*}{\mathrm{g}}_{\mathrm{j}} \left( {t}_{i} \right) = \frac{{a}_{j}}{1+{b}_{j}{\mathrm{e}}^{-{r}_{j}{t}_{i}}} \end{eqnarray*}

where *a*_*j*_, *b*_*j*_ and *r*_*j*_ are the parameters for genotype *j*.

The estimated likelihood of genotype *j* for individual *i* are summed, weighted by the corresponding conditional probability of the QTL genotype, given the adjacent marker and inbred type. Therefore, the functional mapping method formulates the likelihood calculation of the mixture model as:

(3) \begin{eqnarray*}\text{logL} \left( \hat {\Omega } \right) =\sum _{\mathrm{i}=1}^{\mathrm{N}}\log \nolimits \left[ \sum _{\mathrm{j}=1}^{\mathrm{J}}{\mathrm{p}}_{\mathrm{ ij}}{\mathrm{f}}_{\mathrm{j}}({\mathrm{y}}_{\mathrm{i}}) \right] \end{eqnarray*}

where each element p_ij_ indicates the genotypic possibility of subject *i* for gene *j* (QQ, Qq, or qq), ***N*** denotes the individual number in the experimental population, and $\hat {\Omega }$ denotes the variables in this log-likelihood function that include the covariate coefficient, curve parameters for multiple genotypes, and covariance parameters. In Eq. (3), we prefer to use the log-likelihood function.

The likelihood function for the null hypothesis model, in which QTL does not affect the trait, is built as follows,

(4) \begin{eqnarray*}\text{logL} \left( \tilde {\Omega } \right) =\sum _{\mathrm{i}=1}^{\mathrm{N}}\log \nolimits ({\mathrm{f}}_{0} \left( {\mathrm{y}}_{\mathrm{i}} \right) )\end{eqnarray*}

where ${\mathrm{f}}_{0} \left( {\mathrm{y}}_{\mathrm{i}} \right) $ differs from (1) in g _j_ by assuming the same longitudinal curve for all genotypes. $\tilde {\Omega }$ denotes the estimated parameters in this log-likelihood function.

The goal of functional mapping is to compute the log-likelihood ratio (LR) for each QTL, defined by the following equation, and then to choose the significant QTL with a high value of LR.

(5) \begin{eqnarray*}\mathrm{LR}2=-2\log \nolimits \left[ \frac{L \left( \tilde {\Omega } \right) }{L \left( \hat {\Omega } \right) } \right] .\end{eqnarray*}

Intuitively, the null hypothesis H_0_ is that there is no gene that controls the growth process, and the alternative hypothesis H_1_ is that growth processes are different across the QTL genotypes. $\tilde {\Omega }$ and $\hat {\Omega }$ are the maximum likelihood estimates of parameters under the hypotheses H_0_ and H_1_, respectively.

To conduct a log-likelihood ratio test on complicated statistical models like the one used in functional mapping, a permutation test is usually used to derive and to compare against the null distribution (Churchill & Doerge, 1994). Because the permutation test is generally applicable to various models, it is intensive to implement computationally. To address this, we proposed a new approach called the filtering method to improve the computational efficiency of a permutation test. We first quantified the correlation between QTL and the longitudinal data using a genotype-oriented curve clustering method. Then, the QTLs that are highly correlated with the outcome were computed in the improved permutation tests (Wang et al., 2017). As a result, this reduced the amount of computation in permutation tests significantly and sped up the computation for data analysis in functional mapping.

### Package workflow

**Figure 1:**
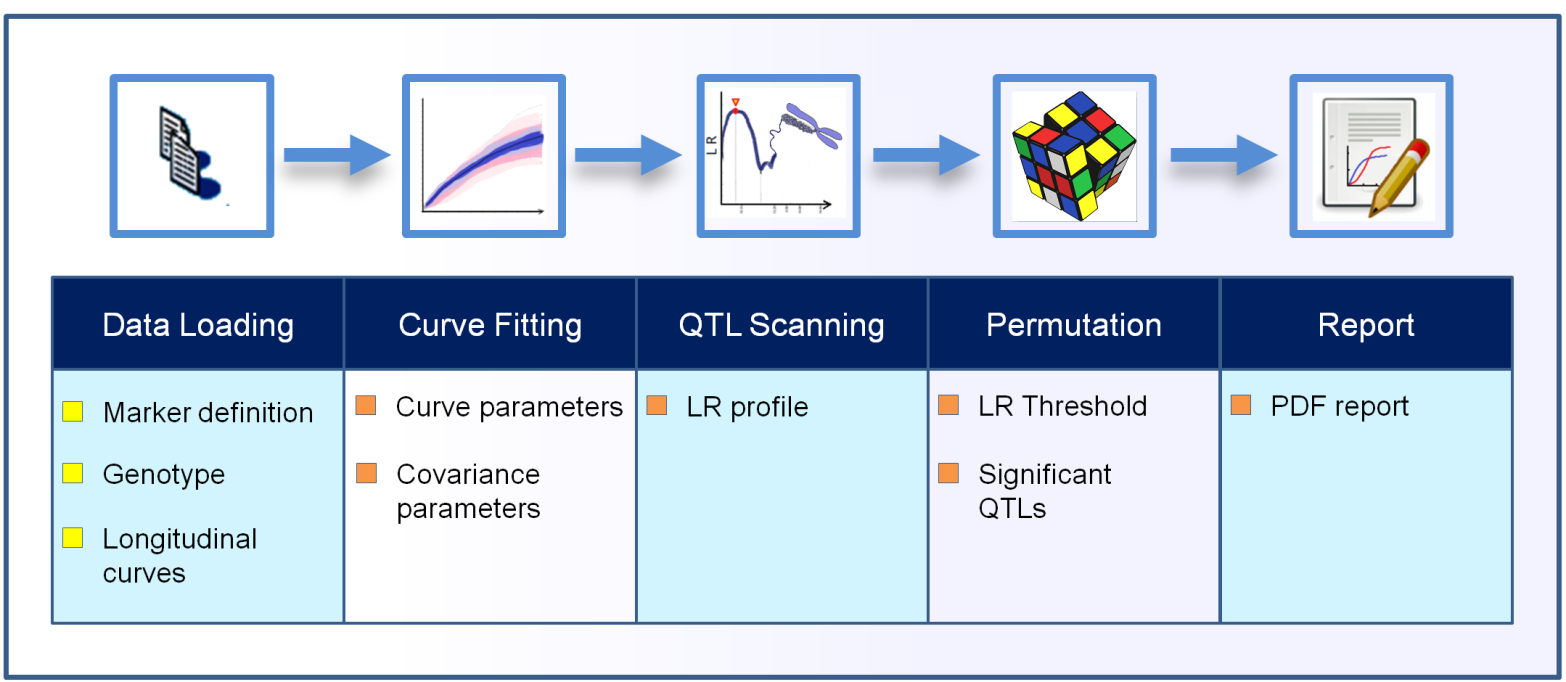
The workflow process in the Funmap2 package. The top row shows the analysis steps and the bottom row shows the corresponding output obtained in each step. The chromosome image is used under Pixabay’s license (https://pixabay.com/en/service/license/).

The *Funmap2* package is an open source package for R with automated data analysis for identifying the significant QTLs for the longitudinal traits that were measured. The *Funmap2* pipeline includes modules for data import, curve fitting, QTL scanning, MLE computation, hypothesis testing, and data visualization (Fig. 1).

The user may either run the entire *Funmap2* workflow in one function call or step-by-step with customization. Often it is more convenient to call the main function *FM2.pipe,* which automatically implements all tasks and outputs the summary information and figures in PDF format. In this function, sub-modules are called successively. Functions include data loading (*FM2.load.data*), data estimation (*FM2.estimate.data*) for curve fitting and covariance selection, QTL scanning (*FM2.qtlscan*), and permutation (*FM.permutation)*, and report generation (*FM2.report*). The typical calling is shown below.

Box 1 # Call the pipeline in parallel computing.

r < - FM2.pipe( file.pheno.csv, NULL, file.geno.csv,file.marker.csv, ”BC”,

curve.type=”logistic”,

covar.type=”auto”,

options=list(n.cores=10) )

*Funmap2* requires users to provide experimental data and to specify several parameters. The phenotype file that contains longitudinal traits, one genotype marker file for the experimental population, and one genetic marker information file are required. A covariate file is optional and is not provided in the above example. In addition to these experimental data, users need to specify the cross type of QTL mapping. Four available types are provided in *Funmap2*: Backcrossing, F2, RIL, and DH. Note that in this automated run, the user may either let *Funmap2* choose the optimal curve type and covariance structure or the user may specify their values as functional arguments.

Alternatively, the data analysis can be conducted by customizing the workflow by running sub-modules of *Funmap2* successively, as illustrated below.

#### Data loading

Input files for *Funmap2*, which include the marker definition file, the genetic marker file, the phenotype file, and the covariate file, should be formatted in CSV format according to the description in the vignette of *Funmap2*. The function *FM2.load.data* reads these data files, checks the correctness of data format and the consistency of individual IDs across all files and, finally, returns an R data object which can be called by the generic functions, such as show, print, plot. Phenotypic traits, and the curves that describe the longitudinal trend (if curve.type is specified) can be visualized by calling the plot function directly on the returned R object.

#### Curve fitting and selection of the covariance matrix

The function *FM2.estimate.data* may be invoked to facilitate the manual selection of curve type and covariance structure. The most commonly used curve function is sigmoid, and it is generally used to characterize growth curves of populations of plants and animals. Curves other than sigmoid, such as those that describe environment-dependent traits for which reaction norms are measured, are also provided. In total, *Funmap2* software implements nine curves in the current version, which include logistic, composite, and those generated by nonparametric methods. When running the automated mode (i.e., curve type is not specified during the data loading), least square curve fitting followed by AIC (Akaike Information criterion) and BIC (Bayesian Information criterion) is used to determine the best curve type.

The internal correlation of traits measured at different longitudinal points are described using the covariance matrix, which is essential in the likelihood calculation. We included a comprehensive set of 13 covariance matrices, which include those employed by SPSS software and first order ante-dependence (Yap, Fan & Wu, 2009). *Funmap2* also implements an automated way to select a covariance matrix using the AIC or MLE method. Users should be cautious of the cost of computational time when over-parametrizing the covariance matrix. The details of curves and covariance matrices are available in the vignette of *Funmap2*.

#### QTL scanning

The function *FM2.qtlscan* estimates the effects of QTLs and parameters that characterize longitudinal trend curves by scanning across all QTL positions. Specifically, the function tests H_0_ and H_1_ hypotheses using the MLE method and calculates LR2 values in Eq. (5) at each QTL marker at every 1 cM position. The MLE method also outputs covariate coefficients, curve parameters and covariance parameters that optimize the log-likelihood. When finished, *FM2.qtlscan*, by default, generates the LR2 profile figure and highlights QTLs with the highest LR2 value for each chromosome. To determine the significant QTLs, the user needs to run the permutation test (see the following section).

#### Permutation test

The distribution of the log-likelihood ratio is difficult to derive in analytical form, especially for complicated distribution functions. To overcome this difficulty, a permutation test is generally used to obtain the null distribution and declare statistical significance of a QTL. One commonly encountered issue with a permutation test, however, is the high cost of computational time, which becomes especially prominent when running the entire genome. The function *FM.permutation* includes two methods to address this issue. The first option is to parallelize the computation in a unix-based operating system provided that multiple processor threads are available. The second option is to apply a filtering method to reduce the computational intensity. This is done by pre-selecting the candidate QTLs that have a high QTL-trait correlation.

#### Report

The resulting objects returned by individual functions contain summary information (by the summary function), and they can be visualized by the plot function. In addition, *Funmap2* includes the function *FM2.report* that can generate reports automatically, which include tiled/overlapping curves that describe the longitudinal trait, LR profile for all chromosomes, multiple LR2 profiles for the significant QTLs, and the curves for the significant QTLs.

## Results

**Figure 2:**
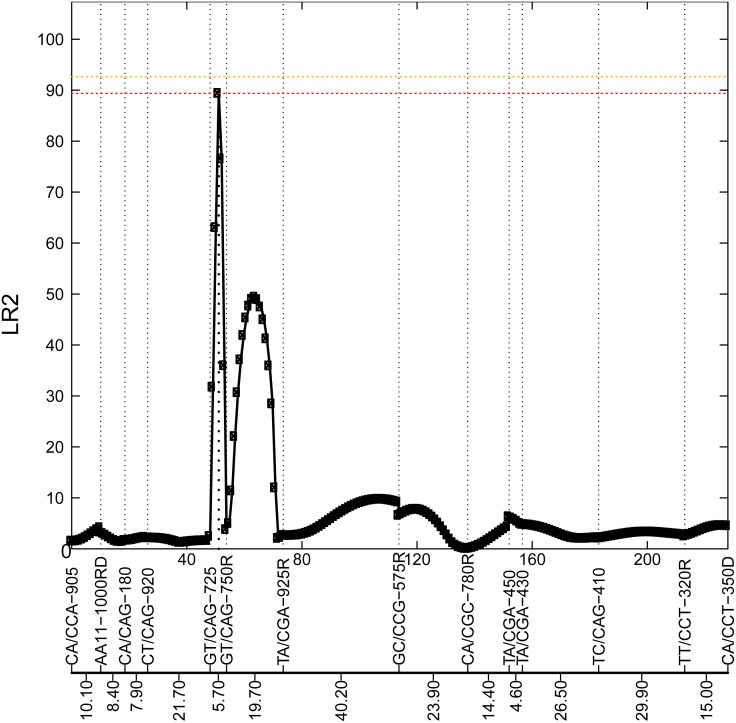
Profile of the likelihood ratio (LR) for the 8th chromosome. This figure shows 14 markers in this chromosome, with their names and genetic distance for each interval highlighted at the bottom. The peak with LR2=89.47 at interval [GT/CAG-725—GT/CAG-750R] is located at 3 cM from GT/CAG-725, which suggests a potential locus for a significant QTL.

**Figure 3:**
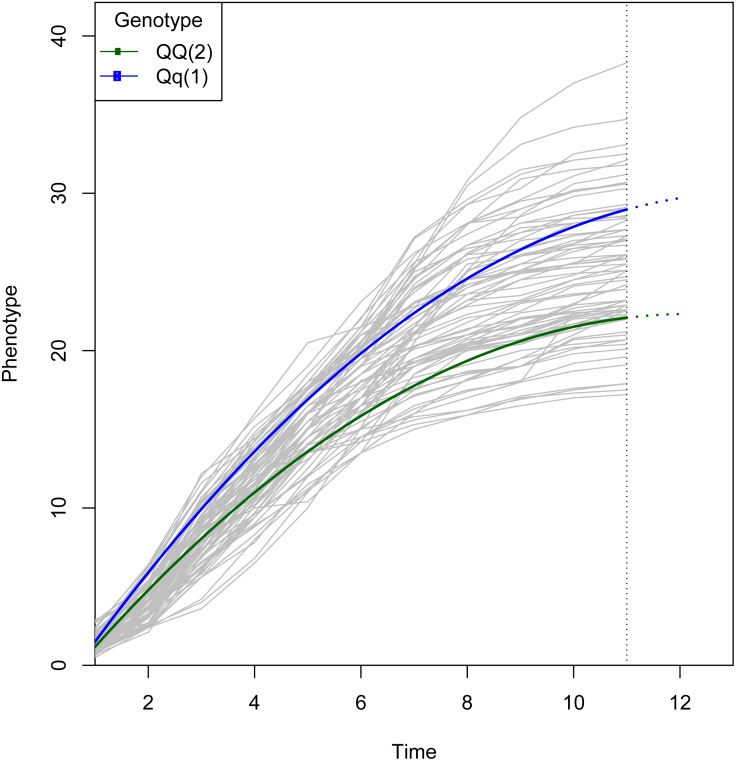
Growth curve for the QTL selected from Fig. 2. The logistic curve was chosen by curve fitting. The curves are colored by genotype. Note that two genotypes show similar growth at early stages, but diverge at later stages, which suggests there is a QTL associated with growth kinetics. The two genotypes have different parameter values in the growth trajectory function defined by g_*j*_ in Eq. (2).

We use a data set from the pre-installed Populus data set (Ma, Casella & Wu, 2002) in this package as an example. The data are composed of 90 backcross individuals with 22 linkage groups and 275 molecular markers. The phenotypic values were measured throughout 11 years. The code for analyzing the data is shown below, and the results for QTL mapping are plotted (Figs. 2 and 3). The computational burden of Funmap2 roughly comes from genome length and permutation. In addition, the computational cost also depends on the sample size and the number of measurements. The analysis of the Populus data set on the whole genome 3611 QTLs with 1000 conventional permutations took 110.2 hr on an Intel(R) Xeon(R) CPU E5-4620 @ 2.6 GHZ computing cluster with 16 cores. This result demonstrates that Funmap2 is computationally intensive for large-scale studies using the conventional permutation method. In order to reduce the computational time of permutation, we proposed a new method that reduces the number of QTLs during the permutation computation because QTLs are highly correlated with the outcome (Wang et al., 2017). In Funmap2, this new permutation can reduce 80% to 95% of the computational cost.

Box 2 # Load the pre-installed data for the example

file.pheno.csv <- system.file(”extdata”,”populus.BC.pheno.csv”, package=”Funmap2”)

file.geno.csv <- system.file(”extdata”,”populus.BC.geno.csv”, package=”Funmap2”)

file.marker.csv <- system.file(”extdata”,”populus.BC.marker.csv”, package=”Funmap2”)

r <- FM2.pipe( file.pheno.csv, NULL, file.geno.csv, file.marker.csv, ”BC”,

curve.type=”auto”, covar.type=”AR1”, options=list (n.cores=10))

## Discussion

Functional mapping models assume that longitudinal traits follow a parametric or non-parametric curve, such as a growth trajectory, Legendre polynomial, or B-Spline (Yang, Wu & Casella, 2009). Under this assumption, the likelihood ratio and QTL effects that are derived from the parameters of a parametric or a non-parametric curve are calculated by the MLE function over all linkage groups. *Funmap2* implements the functional mapping framework with nine curves and 13 covariance structures. Importantly, any new curve functions that are not implemented by *Funmap2* can be imported easily into the package and assembled into the framework of MLE. It has an open architecture, so the longitudinal traits can be fitted to any biological curve.

The longitudinal traits tend to correlate strongly between time points (time-dependent) or reaction norms (environment-dependent). Functional mapping models this internal relation using a covariance matrix, which may increase the statistical power for QTL detection (Ma, Casella & Wu, 2002). Whereas previous publications on functional mapping recommended the use of the most parsimonious covariance matrix (Yap, Fan & Wu, 2009), such as autoregressive, ante-dependence, or autoregressive moving average (Li et al., 2010a), *Funmap2* also provides other covariance matrices implemented in IBM SPSS software, such as Compound Symmetry, Factor Analytic, Huynh-Feldt, and Toeplitz. Although a parsimonious covariance matrix can be efficient computationally, non-parsimonious covariance structures contain more parameters and, hence, richer structures, which may potentially lead to better data fitting while minimizing the pitfall of overfitting when guided by information criteria (Zimmerman et al., 2001).

Since Functional Mapping was proposed in 2002, two programs, *FunMap* (Ma, Wu & Casella, 2004) and *3Funmap* (Tong et al., 2011), have been released. *FunMap* employed 3 curve functions and the first-order autoregressive model to implement a basic framework as a web application which is unavailable. *3FunMap*, a Windows Application in Visual C++, implemented linkage map construction and QTL mapping using the Legendre polynomial curve and the first-order autoregressive model. Although *Funmap2* inherited from same framework, it increased model flexibility and software usability with many new features, such as implementing covariates for each individual, more trajectories and covariance matrices available in the mixture model, parallel computing, and using open source development platform. We believe *Funmap2* is the best choice to map QTL for functional trails so far.

## Conclusions

Studies of QTL mapping for longitudinal traits other than functional mapping are unexpectedly rare, compared to that for QTL mapping of a trait that was measured at a single point. As a result, research on the genetic basis that underlies biological development and gene-environment interaction are greatly limited. *Funmap2* provides a user-friendly way to dissect these problems, and it facilitates the building of precise genotype-phenotype relation models through QTL mapping. In addition to mapped QTLs, estimates from curve functions may also provide insights for the understanding of the genetic, biochemical, and physiological pathways that govern developmental change (Wang et al., 2012). We are making the endeavor to develop a GUI version *Funmap2* to facilitate the extraction and interpretation of data further. At present, *Funmap2* supports experimental populations derived from a cross between two inbred lines, and it is limited to four types: F2, backcross, recombinant inbred lines, and double-haploid populations. Future versions of *Funmap2* will be able to accommodate populations of more diverse structures and even multiple traits, epistasis effects, allometric, and QTL-QTL interaction.

## Additional Information and Declarations

### Data Availability

The following information was supplied regarding data availability: The raw example data (Ma, Casella and Wu, 2002) is provided in the package *Funmap2*, and *Funmap2* is available at https://github.com/wzhy2000/Funmap2. Users can use the example in the package or check the contents in GitHub (https://github.com/wzhy2000/Funmap2/tree/master/Funmap2/inst/extdata).
